# Ocular Surface Changes Associated with Neurological Diseases

**DOI:** 10.3390/medicina61091693

**Published:** 2025-09-18

**Authors:** Reda Zemaitiene, Gigi Gorgadze, Laura Mockaitiene

**Affiliations:** 1 Department of Ophthalmology, Medical Academy, Lithuanian University of Health Sciences, 44037 Kaunas, Lithuania; 2 Faculty of Medicine, Tbilisi State Medical University, Tbilisi 0177, Georgia

**Keywords:** ocular surface, neurological disease, dry eye disease, Alzheimer’s disease, Parkinson’s disease, Guillain-Barré syndrome, trigeminal neuralgia, multiple sclerosis, Charcot–Marie–Tooth disease

## Abstract

Neurological disorders significantly affect ocular surface homeostasis, influencing parameters such as blink rate (BR), tear production, corneal nerve density, and sensitivity. This review summarizes recent findings on ocular surface alterations associated with neurological diseases, including Alzheimer’s disease (AD), Parkinson’s disease (PD), Guillain-Barré syndrome (GBS), trigeminal neuralgia (TN), multiple sclerosis (MS), and Charcot–Marie–Tooth disease (CMT). Notably, ocular manifestations such as reduced BR, decreased tear break-up time (TBUT), impaired tear secretion, and corneal nerve fiber loss are consistently reported. In AD, elevated tear amyloid-beta and tau proteins emerge as promising biomarkers for early disease detection. PD patients frequently experience dry eye symptoms attributed to reduced BR and tear film instability. GBS is linked to lagophthalmos and corneal nerve impairment, potentially leading to severe ocular surface damage. TN demonstrates bilateral ocular surface dysfunction despite unilateral neuropathic symptoms. MS is associated with significant ocular surface alterations, reflecting broader neuroinflammatory and autonomic disturbances. Similarly, CMT patients show reduced corneal sensitivity and tear production, underscoring the systemic nature of neurological impacts. Awareness of these ocular manifestations is essential for improving patient care and guiding future research into ocular biomarkers and targeted therapies.

## 1. Introduction

Dry eye disease (DED), a multifactorial condition affecting the tears and ocular surface, has increasingly been linked to various neurological disorders. DED, which results in symptoms of discomfort, visual disturbance, and tear film instability with potential damage to the ocular surface, is accompanied by increased osmolarity of the tear film and inflammation of the ocular surface [1]. DED can significantly impact visual function and quality of life, as its symptoms often disrupt daily activities such as reading, writing, or prolonged use of display monitors [1]. Prevalence rates of DED vary globally, ranging from about 4.6% in North America up to 47.9% in Africa, with an overall global prevalence estimated at 11.6% with prevalence increasing steadily with age and reaching higher rates in adults over 40 years [2].

Neurological disorders have a significant impact on many systems of the body. Recently, neurologists and ophthalmologists have been focused on disturbances and changes in ocular surface homeostasis in various neurological pathologies [3]. Changes are diverse and can vary from DED to such a complex disease as neurotrophic keratitis (NK) [1].

The interaction between neurological disorders and ocular surface health is multifaceted. It includes immune dysregulation, nerve damage, altered blink dynamics, changes in tear production and composition, autonomic dysfunction, and impaired sensory feedback from the cornea and conjunctiva [1]. Disrupting the integrity of the ocular surface leads to several pathologies of the eye surface, including DED, neurotrophic keratopathy, exposure keratopathy, or infectious keratitis, which can affect vision quality and the overall ocular health [4].

Awareness and understanding of the interactions noted in various neurological disorders are important for the timely diagnosis of ocular diseases and the development of the correct therapeutic and preventive strategy. Moreover, patients with neurological conditions may have reduced awareness of their ocular problems [5], which can further complicate diagnosis and delay effective treatment.

This paper focuses on Alzheimer’s disease, Parkinson’s disease, Guillain-Barré syndrome, trigeminal neuralgia, multiple sclerosis, and Charcot–Marie–Tooth disease, as they often involve cranial nerves essential for ocular health, and are associated with common ocular surface problems like DED.

The purpose of this review is to analyze ocular surface changes, characterized by tear film instability, reduced tear production, and corneal nerve alterations, associated with Alzheimer’s disease, Parkinson’s disease, Guillain-Barré syndrome, trigeminal neuralgia, multiple sclerosis, and Charcot–Marie–Tooth disease.

## 2. Materials and Methods

Our methodology was informed by the Preferred Reporting Items for Systematic Reviews and Meta-Analyses (PRISMA) guidelines [6]. We performed a comprehensive search in PubMed, PubMed Central (PMC), and Web of Science covering the period from January 2020 to March 2025. Given the heterogeneity of study designs and reported outcomes, we conducted a qualitative narrative synthesis rather than a meta-analysis. Search terms included “dry eye disease”, “ocular surface” in combination with “Alzheimer’s disease”, “Parkinson’s disease”, “Guillain-Barré Syndrome”, “Trigeminal neuralgia”, “Multiple Sclerosis”, and “Charcot-Marie-Tooth disease”.

Studies were included if they were original articles, clinical trials, case–control or cohort studies, or case reports evaluating ocular surface changes in the selected neurological disorders, provided they were published in English and available in full text. Reviews, abstracts without accessible full text, non-English publications, and articles unrelated to ocular manifestations were excluded.

Initial screening of titles and abstracts was performed to identify potentially relevant articles. Full texts of eligible studies were subsequently retrieved and reviewed in detail. The selection process involved two independent reviewers who assessed the methodological relevance and scientific quality of each article. In cases of disagreement, consensus was reached through discussion.

**Figure 1:**
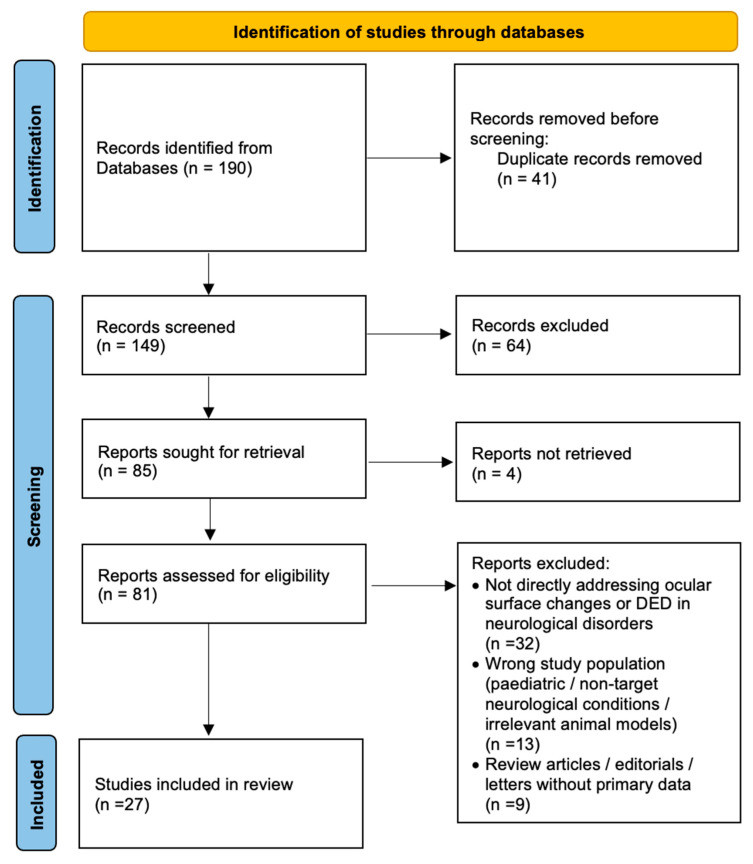
Flowchart of the selection of the different studies used in this article.

The search of the databases resulted in 190 entries, of which 41 duplicates were removed, leaving 149 unique studies. Titles and abstracts were independently screened by two authors, and full texts were reviewed for eligibility. Finally, 27 studies were included in the analysis. From each study, data were extracted on sample size, patient characteristics, diagnostic methods for ocular surface assessment (such as tear break-up time, Schirmer test, corneal confocal microscopy, and impression cytology), and key ocular findings. A detailed selection of different references used in this article is shown in a flowchart (Figure 1).

## 3. Alzheimer’s Disease

Alzheimer’s disease (AD) is the most common neurodegenerative disorder and the leading cause of cognitive impairment in the elderly, accounting for 60–80% of all cases [7]. As for 2023, an estimated 6.7 million Americans aged 65 and above were living with AD [8]. Nowadays, it still remains a growing global health problem with a large impact on both the individual and society.

AD is characterized by abnormal deposits of amyloid-beta and tau proteins, leading to inflammation, which causes neurodegeneration, synaptic dysfunction, and neuronal loss in the hippocampus, cortex, and various lobes [9]. The eye also exhibits several characteristics of AD pathology that are present in the brain. Several ocular findings in AD have been identified, including amyloid precursor protein (APP) and amyloid-beta degradation products in human corneal epithelial cells and fibroblasts, suggesting that APP metabolism and AD-related protein presence in the cornea may serve as potential markers for diagnosing Alzheimer’s disease [10]. Furthermore, alterations in corneal epithelial cells and subbasal nerve fibers have been observed in AD patients [11]. In addition, a variety of structural and functional ocular alterations have been associated with AD progression, such as structural changes in the retina, ocular surface abnormalities, and corneal nerve changes [12].

Gundogan et al. investigated alterations in the corneal subbasal nerve plexus and corneal sensitivity in AD patients [13]. It was found that patients with AD (n = 22) had significantly reduced corneal nerve parameters compared to healthy controls (n = 18): nerve fiber length (NFL) (*p* < 0.001), nerve fiber density (NFD) (*p* < 0.001), and nerve branch density (NBD) (*p* = 0.001) were all markedly lower in the AD group. Corneal sensitivity, measured by esthesiometry, was also significantly decreased in these patients (*p* < 0.001) [13]. These reductions in both nerve structure and function suggest that AD affects peripheral corneal innervation, and these changes correlate with sensory dysfunction, manifesting clinically as reduced corneal sensitivity, impaired tear film stability, and symptoms of DED [13].

Chaitanuwong et al. investigated DED in AD patients (n = 24) compared to cognitively normal controls (n = 39) in their pilot study [14]. Although the prevalence of dry eye was slightly higher in the control group (15%) than in the AD group (13%) based on the Asia Dry Eye Society criteria, the difference was not significant. Also, there were no significant differences in tear break-up time (TBUT) or blink rate (BR) (AD group 30.8 blinks/min vs. control group 35.8 blinks/min) between the groups. The Ocular Surface Disease Index (OSDI) scores were significantly higher in the control group (10.8 (7.2) vs. 12.99 (1.23), *p* < 0.05), suggesting a discrepancy between subjective symptoms and objective signs of dry eye in AD patients [14]. This may be due to reduced corneal sensitivity or impaired symptom perception in those with cognitive decline, potentially leading to underreporting of dry eye symptoms.

A study by D’Antonio et al. found that patients with AD (n = 18) had a significantly lower BR (mean BR: 15.1 blinks/min) compared to healthy controls (n = 20) (mean BR: 20.1 blinks/min; *p* < 0.05) [15]. This reduction suggests involvement of the dopaminergic system in AD, as BR is considered a marker of dopaminergic activity. The findings support the idea that dopaminergic dysfunction may play a role in the neurodegenerative processes underlying AD.

Dehghani et al. reported that patients with mild cognitive impairment (MCI) and dementia had lower sub-basal corneal nerve fiber density (CNFD: 24.5 ± 9.6 fibers/mm^2^ in MCI, 20.8 ± 9.3 in dementia, vs. 32.0 ± 7.5 in controls, *p* < 0.0001), branch density (CNBD: 59.3 ± 35.7 and 53.9 ± 38.7 vs. 90.9 ± 46.5, *p* < 0.0001), and fiber length (CNFL: 17.2 ± 6.5 and 15.8 ± 7.4 vs. 22.9 ± 6.1, *p* < 0.0001). Furthermore, corneal nerve parameters were significantly associated with both cognitive function and functional independence [16].

The main DED signs associated with Alzheimer’s disease are summarized in Table 1.

In AD, ocular surface changes are becoming a promising tool for non-invasive diagnosis of the early state of the disease. Recent studies have identified significant alterations in tear composition in AD patients, such as decreased levels of lysozyme, lipocalin-1, and other lacrimal proteins such as PIP and SCGB2A1, reflecting lacrimal gland dysfunction and ocular surface neuroinflammation [17]. Additionally, elevated tear beta-amyloid levels have shown strong diagnostic potential for early-stage AD, with up to 81% sensitivity and 93% specificity [18].

In addition, the presence of Aβ and tau proteins in tears offers a promising diagnostic marker for AD, particularly in the preclinical or prodromal stages when clinical symptoms may still be subtle. Studies have shown significantly higher levels of Aβ peptides, total tau (*t*-tau), and phosphorylated tau (*p*-tau) in AD patients compared to healthy controls. Notably, *t*-tau levels were significantly elevated in individuals with dementia, mild cognitive impairment, and even subjective cognitive decline, with *p*-values indicating statistical significance (*p* < 0.05) [12].

Firmani et al. highlight the growing clinical interest in ocular-based diagnostics for AD, emphasizing the potential of combining tear biomarkers with structural ocular imaging such as optical coherence tomography (OCT) or OCT–angiography (OCT-A). These methods allow for the assessment of retinal nerve fiber layer thickness, ganglion cell complex parameters, and microvascular vessel density—alterations that correlate with early AD stages, thereby supporting their role in early detection and patient stratification [19].

## 4. Parkinson’s Disease

Parkinson’s disease (PD) is the second most common neurodegenerative disorder, after AD. Incidence of PD is 4 million people worldwide, with an estimated prevalence of 0.3% in industrialized countries [18]. PD primarily affects older adults, as it is rarely seen in patients under 40 years of age [20].

PD is characterized by the progressive degeneration of dopaminergic neurons in the substantia nigra, leading to reduced dopamine levels in the brain and causing the motor symptoms, including tremor, bradykinesia, rigidity, and postural instability, and non-motor symptoms such as cognitive decline, depression, and sleep disturbances [21].

Bradykinesia in the eyes manifests as reduced BR and abnormally widened palpebral fissures [22]. A large multicenter study was conducted by Borm et al. using the Visual Impairment in Parkinson’s Disease Questionnaire (VIPD-Q). Researchers found that symptoms related to dry eye were highly prevalent in patients with PD (n = 848) (63% of PD patients reported ocular surface complaints, including blurry vision, burning sensation, and watery eyes, compared to 24% of controls) (*p* < 0.001). Dry eye symptoms in PD are likely linked to reduced BR and impaired tear film stability due to dopaminergic dysfunction. Moreover, higher VIPD-Q scores were associated with increased risk of falls and decreased quality of life, highlighting the importance of recognizing and treating dry eye and other visual symptoms in PD to support patient safety and well-being [23].

In a cross-sectional survey by Tester et al. of 92 individuals with PD, symptoms related to ocular surface disorders such as blurry vision (46.1%), burning or gritty sensation (34.8%), and mucus or watery eyes (30.3% and 23.6%, respectively) were among the most frequently reported ocular complaints [24]. These symptoms are believed to be related to dopaminergic dysfunction affecting the motor control of blinking. Notably, individuals who reported more frequent ocular symptoms also showed greater difficulty with vision-dependent activities of daily living, as measured by a positive correlation between ophthalmologic symptoms and functional visual performance (r = 0.49, *p* < 0.01) [24].

According to Ungureanu et al., in a study of 40 PD patients and 40 healthy controls, central corneal thickness (CCT) was significantly reduced in PD (538.9 ± 30.9 µm vs. 557.6 ± 26.6 µm, *p* = 0.005), with thinning observed in both the stromal layer and Bowman’s layer (16.8 ± 2.1 µm vs. 18.1 ± 1.9 µm, *p* = 0.02) [24]. Stromal thickness also correlated negatively with H&Y scores (r = −0.50, *p* < 0.05), indicating that as PD severity worsens, corneal thinning becomes more pronounced. The H&Y score system is used to classify PD severity on a scale from 1 to 5, with higher scores indicating more advanced disease and greater motor impairment [25]. In addition to corneal thinning, PD patients exhibit a markedly reduced BR, averaging 12.7 ± 7.4 blinks per minute compared with 21.8 ± 7.4 blinks per minute in healthy controls (*p* < 0.01), with some cases dropping as low as 2–4 blinks per minute versus the normal 15–20 blinks per minute. Tear production is also significantly impaired, with shorter TBUT (<10 s) and reduced Schirmer’s test values (<10 mm/5 min) relative to controls, contributing to ocular surface stress and structural alterations [25,26]. Reduced BR, combined with decreased tear production and poor tear quality, can contribute to a decrease in CCT and significantly (>50%) reduce corneal nerve fiber density [26]. Corneal nerve loss and reduced blinking may reinforce each other: nerve damage impairs the blinking reflex, while decreased blinking exacerbates ocular surface stress, potentially accelerating further nerve damage and creating a vicious circle [26] (Table 1).

A study by Che et al., performed by using corneal confocal microscopy, revealed that patients with PD (n = 65) exhibit significant corneal nerve fiber loss, which correlates with cognitive and motor dysfunction [26]. Compared to healthy controls (n = 30), PD patients had reduced corneal nerve fiber density (CNFD: 25.83 ± 4.73 vs. 34.33 ± 3.78 no./mm^2^, *p* < 0.001), increased nerve branch density (CNBD: 31.77 ± 14.15 vs. 24.58 ± 8.23 no./mm^2^, *p* = 0.011), and shorter nerve fiber length (CNFL: 14.43 ± 3.31 vs. 15.86 ± 2.27 mm/mm^2^, *p* = 0.035). Notably, CNFD was positively associated with cognitive performance as measured by the Montreal Cognitive Assessment (MoCA) score (r = 0.683, *p* < 0.001) and negatively correlated with motor symptom severity on the UPDRS-III (r = −0.481, *p* < 0.001). Among PD patients, those with dementia had the lowest CNFD (20.86 ± 4.67), significantly lower than those with mild cognitive impairment or normal cognition. These findings suggest that corneal nerve alterations reflect both peripheral and central neurodegeneration and may serve as a non-invasive biomarker for monitoring cognitive decline in PD [26].

According to a meta-analysis by Nagino et al., the prevalence of DED is 61.1% (95% CI: 47.4–74.8%) in PD patients, with higher OSDI scores compared to healthy controls. TBUT is notably decreased in PD patients by an average of 3.0 s (95% CI: −4.4 to −1.7 s), indicating marked tear film instability. Schirmer test values also show reduced tear secretion, with mean differences of −4.3 mm without anesthesia and −4.4 mm with anesthesia, confirming decreased baseline and reflex lacrimation. Additionally, CCT was found to be significantly thinner in PD patients by approximately 17.7 µm (95% CI: −21.7 to −13.7 µm). Other diagnostic tests, such as corneal fluorescein staining, rose Bengal staining, and the phenol red thread test, also showed worsened results in PD patients [27].

According to Buzzi et al., PD medications, including anticholinergics, antimuscarinic agents, amantadine, and antidepressants, can also impair tear production by reducing parasympathetic stimulation to the lacrimal glands, leading to decreased aqueous tear secretion [28]. Early studies on untreated, drug-naïve PD patients reported significantly decreased TBUT (4.3 ± 2.1 s vs. 9.7 ± 3.4 s in controls, *p* < 0.01), while Schirmer test values remained within normal limits (14.8 ± 4.5 mm/5 min vs. 15.6 ± 4.7 mm/5 min), suggesting that tear quantity is initially preserved and that early DED in PD arises primarily from qualitative tear film instability [29]. Meibomian gland dysfunction has also been shown to be more severe in PD patients, with gland dropout scores significantly higher than in controls (mean score 1.9 ± 0.6 vs. 1.2 ± 0.4, *p* < 0.05), consistent with autonomic dysfunction affecting the sebaceous glands [29]. This mechanism may also explain the higher prevalence of seborrheic blepharitis frequently observed in PD patients, further aggravating ocular surface irritation and evaporative dry eye [28].

## 5. Gullain-Barré Syndrome

Guillain-Barré Syndrome (GBS) is an autoimmune disease with an estimated incidence of 0.81 to 1.91 new cases per 100,000 persons annually worldwide [30]. The 20% increase in incidence for every 10-year increase in age is also remarkable. Different from other autoimmune diseases, men have a higher risk of developing GBS than women [30]. The syndrome classically manifests as a postinfectious monophasic polyradiculoneuropathy, affecting the peripheral nervous system and causing muscle weakness and paralysis. It is the most common cause of acute flaccid paralysis and is associated with infectious agents such as Campylobacter jejuni, Zika virus, and SARS-CoV-2, which can trigger the immune response leading to nerve damage [31]. The pathophysiology involves an immune-mediated attack on the myelin sheath or axons of peripheral nerves, causing inflammation, demyelination, and impaired nerve signal transmission [30,31,32].

As a result of these neurological pathologies, GBS patients may experience several ocular pathologies. In GBS, involvement of the facial nerve (cranial nerve VII) can lead to reduced BR and lagophthalmos. These impair normal tear distribution, contributing to DED, exposure keratopathy, and punctate epithelial erosions, thereby increasing the risk of corneal ulceration or infection [33]. While blink reflex abnormalities can be observed in GBS patients, a study by Esmaeilian et al. suggests that reduced BR alone is not a reliable diagnostic marker for Guillain-Barré syndrome and should be interpreted alongside other clinical and electrophysiological findings [33]. Additionally, a recent case report by Qin et al., using in vivo confocal microscopy, demonstrated significant loss of corneal subbasal nerve fibers, resulting in reduced corneal sensitivity and neurotrophic keratopathy in patients with GBS [34] (Table 1).

## 6. Trigeminal Neuralgia

Trigeminal neuralgia (TN) is a rare sensory pathology of one or more branches of the trigeminal nerve, characterized by repeated unilateral, short, electric shock-like pains, triggered by harmless stimuli with sudden onset and termination [35]. According to Latorre et al., the prevalence of trigeminal neuralgia (TN) is estimated at 0.3%, with an incidence rate of 12.6 cases per 100,000 person-years. Incidence increases with age, reaching 17.5 cases per 100,000 person-years in individuals aged 60–69 and 25.6 cases in those aged 70 and older [36]. Primary TN is usually caused by vascular compression of the trigeminal nerve root at the dorsal root entry zone (DREZ). Idiopathic TN describes cases with no detectable vascular or structural cause. Secondary TN results from identifiable conditions such as tumors, multiple sclerosis, post-herpetic neuropathy, vascular malformations, trauma, or autoimmune disease [35,37].

A cross-sectional study by Altas et al. investigated ocular surface changes in patients with unilateral TN (n = 24) and found significant alterations in both eyes, despite the pain being localized to one side [38]. TBUT was significantly lower in both the affected (7.0 s) and contralateral eyes (8.0 s) compared to controls (n = 24) (12.5 s), with a median difference of 3.0 s (*p* = 0.001). Conjunctival impression cytology grades were also significantly worse in the TN group compared to the control group (*p* < 0.001). Although Schirmer 1 test values were lower in TN patients (5.0 mm in the affected eyes and 7.0 mm in contralateral eyes vs. 10.0 mm in controls), these differences did not reach statistical significance. OSDI scores were markedly elevated in TN patients (median 30.2) compared to controls (8.3), with a median difference of 20.8 (*p* < 0.001). These findings suggest that TN may lead to bilateral ocular surface dysfunction, highlighting the involvement of neurosensory pathways in the development of dry eye symptoms and epithelial changes, even in the absence of direct pain [38]. Impaired reflex mucin secretion, along with central sensitization or neuroinflammatory mechanisms, offers a plausible explanation for bilateral goblet cell loss in a clinically unilateral neuropathic condition [38].

A study by Xie et al. evaluated ocular surface alterations in patients with NK (n = 26) resulting from trigeminal nerve injury after neurosurgery [39]. Compared to contralateral and healthy control eyes (n = 20), NK eyes showed significantly reduced corneal sensitivity across five corneal regions. In mild NK eyes, sensitivity ranged from 38.5 to 45.9 mm, while in moderate/severe NK eyes, it was drastically lower, ranging from 7.8 to 15.6 mm (*p* < 0.001). Sub-basal corneal nerve density in moderate/severe NK eyes was significantly reduced, averaging 5013.89 ± 2720.11 µm/mm^2^. Additionally, tear film stability was impaired in mild NK eyes, with first non-invasive tear breakup time (NITBUT-f) averaging 4.00 ± 1.46 s versus 7.39 ± 2.29 s in contralateral eyes and 7.42 ± 2.89 s in healthy controls (*p* < 0.001). BRs were also altered, with a significantly higher partial blinking rate (PBR: 0.79 ± 0.29 blinks/min) and fewer total blinks (5.76 ± 3.40 blinks/min) compared to controls (PBR: 0.21 ± 0.23, total blinks: 11.95 ± 4.68) (*p* < 0.001; *p* < 0.001). These findings also conclude that trigeminal nerve injury leads to bilateral ocular surface dysfunction, even in unilateral disease, with severity closely tied to corneal sensory loss and facial nerve involvement [39] (Table 1).

## 7. Multiple Sclerosis

Multiple sclerosis (MS) is the most common non-traumatic disabling chronic autoimmune neurological disease of young adults. MS affects 2.3 million people worldwide and is often diagnosed between the ages of 20 to 50 years [40,41]. Females are more frequently affected. Pathogenesis includes immune, environmental, and genetic factors. The main symptoms include focal weakness, vision impairment, bowel and bladder dysfunction, and cognitive impairment [40,41].

A cross-sectional study by Belviranli et al. comparing 33 patients with MS to 33 healthy controls revealed significant ocular surface dysfunction in the MS group [42]. MS patients had markedly reduced Schirmer test values (8.45 ± 5.75 mm) and TBUT (8.12 ± 3.16 s) compared to controls (17.36 ± 10.89 mm and 13.06 ± 4.23 s, respectively; *p* < 0.001). Subjective symptoms of dry eye, assessed using the OSDI questionnaire, were also significantly higher in MS patients (36.36 ± 19.19) vs. controls (13.70 ± 15.36; *p* < 0.001). Conjunctival impression cytology (CIC) grades were significantly elevated in the MS group (1.48 ± 0.71 vs. 0.39 ± 0.56; *p* < 0.001), indicating more advanced squamous metaplasia and goblet cell loss. Notably, 42.4% of MS patients had CIC grades 2–3, compared to just 3.3% in the control group. These findings demonstrate that MS is associated with both objective and subjective markers of DED, as well as cellular changes in the ocular surface, supporting the need for routine ophthalmologic evaluation in this population [42].

A cross-sectional case–control study by Sorkhabi et al. assessed the prevalence and severity of DED in patients with MS compared to healthy individuals [43]. Among the 100 MS patients studied, significantly more had abnormal tear function based on Schirmer 1 (12.2 ± 4.1 mm vs. 15.2 ± 4.6 mm; *p* = 0.01), Schirmer 2 (7.8 ± 3.7 mm vs. 10.1 ± 4.4 mm; *p* = 0.01), tear meniscus height (0.4 ± 0.1 mm vs. 0.5 ± 0.2 mm; *p* = 0.02), and TBUT (11.0 ± 5.3 s vs. 14.5 ± 4.6 s; *p* = 0.01) compared to healthy controls (n = 100) (Table 1). The Dry Eye Scoring System (DESS) also revealed higher symptom severity in MS patients (4.7 ± 3.5 vs. 2.6 ± 2.1; *p* = 0.01), with 46% of MS patients reporting symptoms. Furthermore, DED severity correlated with greater disability, particularly in those with EDSS scores above 4. These findings show the inflammatory nature of MS and its impact on tear film function, highlighting the need for early ocular surface assessment in MS patients to prevent further complications and preserve quality of life [43].

## 8. Charcot–Marie–Tooth Disease

Charcot–Marie–Tooth disease (CMT) is the most common inherited neurological disorder that causes peripheral neuropathy—progressive muscle weakness, atrophy, and loss of sensation in the extremities, with a prevalence of 1/2500 [44]. It typically onsets in childhood, and weakness progresses slowly and causes muscular atrophy of the feet and legs, possibly including the depression of tendon reflexes and slight to moderate distal sensory impairment [44].

Ocular surface involvement in CMT disease has emerged as an important yet underrecognized clinical feature, particularly in subtypes associated with MFN2 mutations. While CMT is primarily known for its peripheral neuropathic manifestations, recent studies and case reports have described atypical ocular findings such as congenital cataracts, severe astigmatism, and DED [45].

A study in 26 CMT patients using corneal confocal microscopy demonstrated significantly reduced CNFD (21.3 ± 7.1 fibers/mm^2^ vs. 32.5 ± 6.8 in controls, *p* < 0.001), CNFL (14.2 ± 4.9 mm/mm^2^ vs. 22.8 ± 5.7, *p* < 0.001), and CNBD (41.5 ± 18.7 vs. 76.9 ± 22.1 branches/mm^2^, *p* < 0.001), reflecting impaired sub-basal nerve integrity [46]. Non-contact esthesiometry further revealed reduced corneal sensitivity (mean threshold 0.42 ± 0.16 mbar vs. 0.19 ± 0.07 mbar in controls, *p* < 0.01), suggesting functional as well as structural impairment of corneal innervation [46]. Although DED is not a well-documented ocular manifestation of CMT, it remains a possible symptom of CMT, as it affects the nerves throughout the body, and the cornea is among the most densely innervated tissues [46] (Table 1).

A case report by Aldihan et al. described a 28-year-old woman with CMT who developed severe DED, resulting in a central corneal perforation following minor trauma [47]. Extensive evaluation ruled out autoimmune causes of DED, and genetic testing confirmed CMT with a homozygous MPV17 mutation. The patient had a Schirmer II test result of less than 1 mm, indicating severely impaired tear production. Her condition required intensive management, including corneal gluing, and ultimately a surgical patch graft using a mini-Descemet stripping automated endothelial keratoplasty (mini-DSAEK) technique, amniotic membrane, and fibrin glue. This case highlights that severe DED may be an underrecognized ocular manifestation of CMT, potentially linked to trigeminal nerve involvement and reduced corneal sensation [47].

## 9. Conclusions

Ocular surface changes, including tear film instability, reduced tear production, and corneal nerve changes, are consistently reported in patients with Alzheimer’s disease, Parkinson’s disease, Guillain-Barré syndrome, trigeminal neuralgia, multiple sclerosis, and Charcot–Marie–Tooth disease. Recognizing these manifestations is essential for a timely diagnosis and management of ocular complications in neurological disorders. Future research should focus on validating ocular biomarkers and refining non-invasive diagnostic tools.

## 10. Future Directions

Future research is best focused on studying the underlying pathogenic mechanisms by which different neurological diseases affect the ocular surface. It is essential to recognize that these neurological conditions can significantly impact both the ocular surface and patients’ subjective ocular symptoms, which should not be overlooked but closely monitored to improve overall patient health and quality of life.

Based on the analysis of the current literature, the following ocular surface biomarkers and parameters (Table 2) are proposed as potential tools for the early diagnosis and monitoring of various neurodegenerative and neurological disorders. These markers reflect changes in tear composition, corneal nerve integrity, and ocular surface function that correlate with disease presence and progression.

To improve our understanding in this field, further larger-scale studies are essential, using consistent testing methods and including advanced tools like in vivo confocal microscopy or tear analysis, which will help us better understand all of the underlying pathogenic mechanisms. Moreover, identification and validation of ocular surface biomarkers with high specificity and sensitivity remain a priority; as such, non-invasive markers could contribute to earlier detection, more accurate disease stratification, and potentially improved clinical outcomes through timely intervention.

## Figures and Tables

**Table 1:** Main Dry Eye Disease (DED) signs associated with neurological disorders.

Neurological Disorder	Main DED Signs
Alzheimer’s Disease (AD)	Decreased BR, Tear Film Instability, Reduced Tear Secretion, Increased Tear Osmolarity, Corneal Hypoesthesia, Corneal Nerve Dysfunction.
Parkinson’s Disease (PD)	Decreased BR, Tear Film Instability, Decreased TBUT, Reduced Tear Secretion, Corneal Nerve Fiber Density Loss, Meibomian Gland Dysfunction, Seborrheic Blepharitis.
Guillain-Barré Syndrome (GBS)	Decreased BR, Lagophthalmos, Reduced Tear Distribution, Reduced Tear Production, Risk of Corneal Infections.
Trigeminal Neuralgia (TN)	Reduced TBUT, Bilateral Goblet Cell Loss, High-Grade Squamous Metaplasia, Conjunctival Epithelial Changes.
Multiple Sclerosis (MS)	Loss of Conjunctival Goblet Cells, Autonomic Dysfunction Affecting Tear Production, Corneal Nerve Fiber Density Loss.
Charcot–Marie–Tooth Disease (CMT)	Reduced Corneal Sensitivity, Corneal Nerve Fiber Loss, Decreased Tear Production, Tear Film Instability.

BR—blinking rate, TBUT—tear break-up time.

**Table 2:** Proposed ocular surface biomarkers for early detection and monitoring of neurological diseases.

Biomarker/Parameter	Description	Associated Neurological Diseases	Diagnostic/Clinical Relevance
Beta-amyloid peptides (Aβ)	Elevated levels in tear fluid	Alzheimer’s disease	Early detection and monitoring of disease progression
Tau proteins (total tau, phosphorylated tau)	Increased levels in tears	Alzheimer’s disease	Correlates with cognitive decline and disease severity
Lacrimal gland proteins	Decreased levels of lysozyme, lipocalin-1, PIP, SCGB2A1 in tears	Alzheimer’s disease	Indicates lacrimal gland dysfunction and ocular surface neuroinflammation
Tear Break-Up Time (TBUT)	Reduced tear film stability	Parkinson’s disease, Multiple sclerosis, Trigeminal neuralgia	Reflects impaired tear dynamics and ocular surface dysfunction
Corneal Nerve Fiber Density (CNFD)	Reduced density of corneal subbasal nerve fibers (measured by confocal microscopy)	Alzheimer’s disease, Parkinson’s disease, Guillain-Barré syndrome, Charcot-Marie-Tooth disease	Marker of peripheral nerve degeneration, correlated with disease severity
Corneal Sensitivity	Decreased corneal sensitivity (measured by esthesiometry)	Alzheimer’s disease, Trigeminal neuralgia, Guillain-Barré syndrome, CMT	Reflects neurotrophic changes and risk of ocular surface complications

## Data Availability

No new data were created or analyzed in this study. Data sharing is not applicable to this article.

## Abbreviations

The following abbreviations are used in this manuscript:

BR	Blink Rate
TBUT	Tear Break-Up Time
DED	Dry Eye Disease
NK	Neurotrophic Keratitis
AD	Alzheimer’s Disease
PD	Parkinson’s Disease
H&Y	Hoehn and Yahr
CNFD	Corneal Nerve Fiber Density
OSDI	Ocular Surface Disease Index
GBS	Guillain-Barré Syndrome
TN	Trigeminal Neuralgia
DREZ	Dorsal Root Entry Zone
CIC	Conjunctival Impression Cytology
MS	Multiple Sclerosis
CMT	Charcot–Marie–Tooth Disease

## References

[1] Bron A.J., de Paiva C.S., Chauhan S.K., Bonini S., Gabison E.E., Jain S., Knop E., Markoulli M., Ogawa Y., Perez V. (2017). TFOS DEWS II Pathophysiology Report. Ocul. Surf..

[2] Papas E.B. (2021). The Global Prevalence of Dry Eye Disease: A Bayesian View. Ophthalmic Physiol. Opt..

[3] Roda M., Ciavarella C., Giannaccare G., Versura P. (2020). Biomarkers in Tears and Ocular Surface: A Window for Neurodegenerative Diseases. Eye Contact Lens.

[4] Kaur S., Sohnen P., Swamynathan S., Du Y., España E.M., Swamynathan S.K. (2023). Molecular Nature of Ocular Surface Barrier Function, Diseases That Affect It, and Its Relevance for Ocular Drug Delivery. Ocul. Surf..

[5] Borm C.D.J.M., Werkmann M., de Graaf D., Visser F., Hofer A., Peball M., Smilowska K., Putz D., Seppi K., Poewe W. (2022). Undetected Ophthalmological Disorders in Parkinson’s Disease. J. Neurol..

[6] Page M.J., McKenzie J.E., Bossuyt P.M., Boutron I., Hoffmann T.C., Mulrow C.D., Shamseer L., Tetzlaff J.M., Akl E.A., Brennan S.E. (2021). The PRISMA 2020 statement: An updated guideline for reporting systematic reviews. BMJ.

[7] Testo A.A., Roundy G., Dumas J.A. (2025). Cognitive Decline in Alzheimer’s Disease. Curr. Top. Behav. Neurosci..

[8] Alzheimer’s Association (2023). 2023 Alzheimer’s Disease Facts and Figures. Alzheimers Dement..

[9] Busche M.A., Hyman B.T. (2020). Synergy between Amyloid-β and Tau in Alzheimer’s Disease. Nat. Neurosci..

[10] Choi S.I., Lee B., Woo J.H., Jeong J.B., Jun I., Kim E.K. (2019). APP Processing and Metabolism in Corneal Fibroblasts and Epithelium as a Potential Biomarker for Alzheimer’s Disease. Exp. Eye Res..

[11] Dehghani C., Frost S., Jayasena R., Masters C.L., Kanagasingam Y. (2018). Ocular Biomarkers of Alzheimer’s Disease: The Role of Anterior Eye and Potential Future Directions. Investig. Ophthalmol. Vis. Sci..

[12] Romaus-Sanjurjo D., Regueiro U., López-López M., Vázquez-Vázquez L., Ouro A., Lema I., Sobrino T. (2022). Alzheimer’s Disease Seen through the Eye: Ocular Alterations and Neurodegeneration. Int. J. Mol. Sci..

[13] Gündoğan A.O., Oltulu R., Belviranlı S., Tezcan A., Adam M., Mirza E., Altaş M., Okka M. (2024). Corneal Innervation Changes in Alzheimer’s: Implications for Sensory Dysfunction. Int. Ophthalmol..

[14] Chaitanuwong P., Jariyakosol S., Apinyawasisuk S., Hirunwiwatkul P., Lawanlattanagul H., Hemrungrojn S., Chongpison Y. (2023). Changes in Ocular Biomarkers from Normal Cognitive Aging to Alzheimer’s Disease: A Pilot Study. Eye Brain.

[15] D’Antonio F., Bartolo M.I., Ferrazzano G., Monti M.S., Imbriano L., Trebbastoni A., Berardelli A., Conte A. (2021). Blink Rate Study in Patients with Alzheimer’s Disease, Mild Cognitive Impairment and Subjective Cognitive Decline. Curr. Alzheimer Res..

[16] Dehghani C., Frost S., Jayasena R., Fowler C., Masters C.L., Kanagasingam Y., Jiao H., Lim J.K.H., Chinnery H.R., Downie L.E. (2020). Morphometric Changes to Corneal Dendritic Cells in Individuals with Mild Cognitive Impairment. Front. Neurosci..

[17] Kärkkäinen V., Saari T., Rusanen M., Uusitalo H., Leinonen V., Thiede B., Kaarniranta K., Koivisto A.M., Utheim T.P. (2025). Neuroinflammation Markers in Tear Fluid of Mild Alzheimer’s Disease. J. Mol. Neurosci..

[18] Gharbiya M., Visioli G., Trebbastoni A., Albanese G.M., Colardo M., D’Antonio F., Segatto M., Lambiase A. (2023). Beta-Amyloid Peptide in Tears: An Early Diagnostic Marker of Alzheimer’s Disease Correlated with Choroidal Thickness. Int. J. Mol. Sci..

[19] Firmani G., Salducci M., Testa F., Covelli G.P., Sagnelli P., Lambiase A. (2024). Ocular Biomarkers in Alzheimer’s Disease: Insights into Early Detection through Eye-Based Diagnostics—A Literature Review. Clin. Ter..

[20] Simon D.K., Tanner C.M., Brundin P. (2020). Parkinson Disease Epidemiology, Pathology, Genetics, and Pathophysiology. Clin. Geriatr. Med..

[21] Hayes M.T. (2019). Parkinson’s Disease and Parkinsonism. Am. J. Med..

[22] Armstrong M.J., Okun M.S. (2020). Diagnosis and Treatment of Parkinson Disease: A Review. JAMA.

[23] Borm C.D.J.M., Visser F., Werkmann M., de Graaf D., Putz D., Seppi K., Poewe W., Vlaar A.M.M., Hoyng C., Bloem B.R. (2020). Seeing Ophthalmologic Problems in Parkinson Disease: Results of a Visual Impairment Questionnaire. Neurology.

[24] Tester N.J., Liu C.J., Shin Y.C., Wagle Shukla A. (2023). Visual Dysfunction and Occupational Performance in Persons with Parkinson’s Disease. Am. J. Occup. Ther..

[25] Ungureanu L., Chaudhuri K.R., Diaconu S., Falup-Pecurariu C. (2023). Dry Eye in Parkinson’s Disease: A Narrative Review. Front. Neurol..

[26] Che N.-N., Jiang Q.-H., Ding G.-X., Chen S.-Y., Zhao Z.-X., Li X., Malik R.A., Ma J.-J., Yang H.-Q. (2021). Corneal Nerve Fiber Loss Relates to Cognitive Impairment in Patients with Parkinson’s Disease. npj Parkinsons Dis..

[27] Nagino K., Sung J., Oyama G., Hayano M., Hattori N., Okumura Y., Fujio K., Akasaki Y., Huang T., Midorikawa-Inomata A. (2022). Prevalence and Characteristics of Dry Eye Disease in Parkinson’s Disease: A Systematic Review and Meta-Analysis. Sci. Rep..

[28] Buzzi M., Giannaccare G., Cennamo M., Bernabei F., Rothschild P.R., Vagge A., Scorcia V., Mencucci R. (2022). Ocular Surface Features in Patients with Parkinson Disease on and Off Treatment: A Narrative Review. Life.

[29] Yaisawang S., Kasetsuwan N., Reinprayoon U., Sringean J., Bhidayasiri R., Pongpirul K. (2020). Dry Eye and Parkinson’s Disease: A Literature Review [abstract]. Mov. Disord..

[30] Shahrizaila N., Lehmann H.C., Kuwabara S. (2021). Guillain-Barré Syndrome. Lancet.

[31] Bellanti R., Rinaldi S. (2024). Guillain-Barré Syndrome: A Comprehensive Review. Eur. J. Neurol..

[32] Leonhard S.E., Cornblath D.R., Endtz H.P., Sejvar J.J., Jacobs B.C. (2020). Guillain-Barré Syndrome in Times of Pandemics. J. Neurol. Neurosurg. Psychiatry.

[33] Ansary B., Ghaderi Ehsanpour M., Esmaeilian S. (2023). Blink Reflex Value in the Early Diagnosis of Guillain-Barré Syndrome. Neuro Res..

[34] Qin D., Wang L., Peng X., Yin H. (2024). Guillain-Barré Syndrome with Associated Bilateral Neurotrophic Keratopathy. J. Neuroophthalmol..

[35] Khawaja S.N., Scrivani S.J. (2023). Trigeminal Neuralgia. Dent. Clin. N. Am..

[36] Latorre G., González-García N., García-Ull J., González-Oria C., Porta-Etessam J., Molina F.J., Guerrero-Peral A.L., Belvís R., Rodríguez R., Bescós A. (2023). Diagnosis and Treatment of Trigeminal Neuralgia: Consensus Statement from the Spanish Society of Neurology’s Headache Study Group. Neurologia (Engl. Ed.).

[37] Xu R., Xie M.E., Jackson C.M. (2021). Trigeminal Neuralgia: Current Approaches and Emerging Interventions. J. Pain Res..

[38] Altaş M., Oltulu P., Uca A.U., Belviranlı S., Gündoğan A.O., Mirza E., Oltulu R. (2022). Impact of Unilateral Trigeminal Neuralgia on Bilateral Ocular Surface Alterations. Headache.

[39] Xie C., Liu B., Zhao X., He Q., Liu L., Wei R. (2023). Characteristics of the Ocular Surface in Neurotrophic Keratitis Induced by Trigeminal Nerve Injury Following Neurosurgery. Int. Ophthalmol..

[40] Dobson R., Giovannoni G. (2019). Multiple Sclerosis—A Review. Eur. J. Neurol..

[41] Haki M., Al-Biati H.A., Al-Tameemi Z.S., Ali I.S., Al-Hussaniy H.A. (2024). Review of Multiple Sclerosis: Epidemiology, Etiology, Pathophysiology, and Treatment. Medicine.

[42] Belviranlı S., Oltulu P., Uca A.U., Gündoğan A.O., Mirza E., Altaş M., Turk N., Oltulu R. (2022). Conjunctival Impression Cytology and Tear Film Parameters in Patients with Multiple Sclerosis. Int. Ophthalmol..

[43] Sorkhabi R., Khavandi S., Ayromlou H., Ahoor M.H., Mohammadkhani M., Tabibzadeh E. (2024). Frequency of Dry Eye Syndrome in Patients with Multiple Sclerosis: A Cross-Sectional Case Control Study. J. Res. Clin. Med..

[44] Barreto L.C., Oliveira F.S., Nunes P.S., de França Costa I.M., Garcez C.A., Goes G.M., Neves E.L., de Souza Siqueira Quintans J., de Souza Araújo A.A. (2016). Epidemiologic Study of Charcot-Marie-Tooth Disease: A Systematic Review. Neuroepidemiology.

[45] Nan H., Hata T., Fukao T., Chen W., Kurita T., Natori T., Takiyama Y. (2021). MFN2-Related Charcot-Marie-Tooth Disease with Atypical Ocular Manifestations. Intern. Med..

[46] Moshirfar M., Tukan A.N., Bundogji N., Ronquillo Y.C. (2022). Charcot–Marie–Tooth disease and implications on corneal refractive surgery. Ophthalmol. Ther..

[47] Aldihan K.A., AlRashedi M.J., Bin Helayel H., AlMutlak M., Hameed S.T. (2023). Severe dry eye disease in Charcot–Marie–Tooth disease: A comprehensive case report. Am. J. Case Rep..

